# Soluble Sema4D From γδ T Cells Exerts Osteoblast Inhibition via Plexin‐B/mTOR Signalling Contributing to Pathogenesis of Bisphosphonate‐Related Osteonecrosis of the Jaws

**DOI:** 10.1111/cpr.70114

**Published:** 2025-09-04

**Authors:** Lingling Ou, Shijia Qiao, Zhuoyi Liao, Xiner Tan, Hui Huang, Zhiyan Zhou, Ruhui Luo, Weijun Zeng, Yan Yang, Zhongxuan Zhang, Jingchen Chen, Shengli Wang, Yiqin Jiang, Jianlei Hao, Yuqin Shen, Longquan Shao

**Affiliations:** ^1^ School and Hospital of Stomatology, Guangdong Engineering Research Center of Oral Restoration and Reconstruction Guangzhou Medical University Guangzhou China; ^2^ Stomatological Hospital, School of Stomatology Southern Medical University Guangzhou China; ^3^ Institute of Mass Spectrometry and Atmospheric Environment Jinan University Guangzhou China; ^4^ Guangdong Second Traditional Chinese Medicine Hospital Guangzhou China; ^5^ The Second Affiliated Hospital of Zhejiang University School of Medicine, Jinan University Guangzhou China; ^6^ The Biomedical Translational Research Institute Jinan University Guangzhou China; ^7^ Department of Otolaryngology Shenzhen Longgang Otolaryngology Hospital & Shenzhen Otolaryngology Research Institute Shenzhen China; ^8^ Guangdong Provincial Key Laboratory of Construction and Detection in Tissue Engineering Southern Medical University Guangzhou China

**Keywords:** BRONJ, gel‐BG@ab, MMP3, Sema4D, γδ T cells

## Abstract

Bisphosphonate‐related osteonecrosis of the jaw (BRONJ) is a severe complication in patients undergoing long‐term bisphosphonate therapy, while our knowledge on the pathogenesis of BRONJ is far from sufficient. Gamma delta (γδ) T cells predominantly distribute in mucosal tissues and play an important role in both immune modulation and bone metabolism; however, the mechanism of γδ T cells in the pathogenesis of BRONJ has not been elucidated. Here, we induced BRONJ‐like lesions in wild‐type (WT) and T‐cell receptor delta‐deficient (TCRδ^−/−^) mice via intraperitoneal zoledronate injection. Our findings revealed that γδ T cells infiltrating BRONJ lesions suppressed osteoblast differentiation, whereas γδ T cell depletion in TCRδ^−/−^ mice restored osteogenic function and significantly reduced BRONJ lesion incidence. Mechanistically, we identified matrix metalloproteinase 3 (MMP3) secreted by activated γδ T cells as a critical enzyme cleaving membrane‐bound Sema4D (mSema4D) into soluble Sema4D (sSema4D). This cleavage product bound to Plexin‐B1/2 receptors on osteoblasts, activating the mTOR signalling pathway to inhibit osteogenic differentiation (ALP/Runx2 downregulation). To promote the repair of BRONJ lesions, we engineered a dual‐functional composite hydrogel (Gel‐BG@ab) combining PLGA‐PEG‐PLGA with mesoporous bioactive glass (BG) and anti‐Sema4D antibodies. This composite hydrogel achieved sustained antibody release, effectively neutralising sSema4D, restoring osteoblast activity and reducing the formation of BRONJ‐like lesions in vivo*.* This study provides evidence of MMP3‐Sema4D‐Plexin‐B1/2/mTOR crosstalk in BRONJ and introduces a targeted biomaterial strategy to disrupt pathogenic feedback loops. The Gel‐BG@ab is the integration of immunomodulation and regenerative medicine, providing both theoretical and technical insights for the immune‐material combination therapy of BRONJ.

## Graphical abstract

Schematic summarizing the pathway of sSema4D from γδ T cells inhibiting osteoblast differentiation in BRONJ.

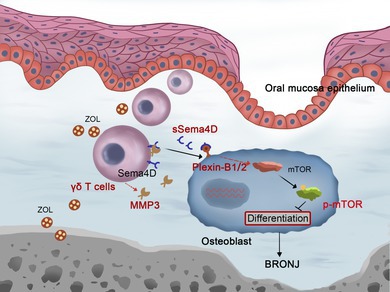

## 1 Introduction

Bisphosphonates are widely used for the treatment of osteoporosis [1], hypercalcemia caused by malignant tumours, breast cancer bone metastasis and pathological fractures, etc. [2], and have significant therapeutic effects [3]. However, the cumulative incidence of bisphosphonate‐related osteonecrosis of the jaw (BRONJ) has reached 9%–12%, causing symptoms such as gingival ulceration, necrotic bone formation, and bone exposure, resulting in significant pain [4, 5, 6]. Despite several identified risk factors [7, 8, 9], the underlying mechanisms remain largely unknown, and there is currently no specific targeting drug [10]. Exploring the potential pathophysiologic mechanisms of BRONJ and finding effective treatments have become crucial issues.

Research indicates that local immune and inflammatory responses disorder caused by bisphosphonates, inducing the occurrence and/or progression of BRONJ [11, 12, 13]. γδ T cells are innate‐adaptive immune intermediaries, predominantly distributed in mucosal tissues [14, 15, 16]. γδ T cells are central regulators in bone immune microenvironments and an inflammatory amplification focus due to their unique immunoregulatory and bone metabolism‐modulating roles [17, 18, 19]. It has been suggested that γδ T cells contribute to the occurrence and development of BRONJ [20, 21, 22], but the specific mechanisms have not been determined. Our studies and other research have shown that bisphosphonates (Zoledronic acid, ZOL) can induce γδ T cell activation and expansion in vitro, promoting cytokine release [23, 24, 25]. The continuous or aberrant activation of γδ T cells promotes the secretion of cytokines such as IFN‐γ, IL‐17, IL‐13, IL‐22, and IL‐23, leading to tissue damage [26, 27].

Reduced bone turnover and new bone formation are the main hypotheses regarding the pathogenesis of BRONJ [28, 29, 30]. In our study, we found that γδ T cells were massively recruited to the extraction sockets in BRONJ‐like mice, and the depletion of γδ T cells alleviated the inhibition of new bone formation. However, how γδ T cells affected the ability of osteoblasts to promote BRONJ development had not been reported. Previous studies have primarily focused on the static expression of Sema4D; we found that γδ T cells highly expressed Sema4D and underwent dynamic conversion between membrane‐bound and soluble forms in this study. We further elucidated that the matrix metalloproteinase 3 (MMP3) cleaved membrane‐bound Sema4D on activated γδ T cells, and the cleaved soluble Sema4D bound to Plexin‐B1/2 on osteoblasts, activating the mTOR signalling pathway, subsequently inhibiting the differentiation of osteoblasts. It uncovered a process of membrane Sema4D cleavage leading to the soluble Sema4D release to receptor binding. Thus, we constructed a composite hydrogel of Gel‐BG@ab (PLGA‐PEG‐PLGA‐BG@αSema4D) to treat BRONJ lesions by controlled releasing anti‐Sema4D antibody. Gel‐BG@ab promoted good healing of BRONJ‐like lesions and regeneration of the alveolar bone and soft tissues, which achieved controlled release and localised delivery of anti‐Sema4D antibodies, overcoming the limitations of systemic administration, and providing both theoretical and technical insights for the immune‐material combination therapy of BRONJ.

## 2 Materials and Methods

### 2.1 Animals

Sprague–Dawley (SD) rats (aged 8–10 weeks, obtained from Vital River Laboratory, Beijing, China), C57BL/6J WT mice and TCRδ^−/−^ mice (aged 8–10 weeks, obtained from Huawei Testing Co. Ltd., Guangdong, China) were used in this study. The animals were housed in SPF facilities, and all experimental procedures were performed in accordance with the Institutional Animal Care and Use Committee approval (Ethics no. 20210429‐02, 202301004).

### 2.2 Preparation and Characterisation of the Composite Hydrogel (Gel‐BG@Ab)

Mesoporous bioactive glass (BG, 104014) was obtained from XFNANO Materials Tech Co. Ltd. (Nanjing, China). BG bound with antibodies (BG@ab) was obtained following immersion in a solution of anti‐Sema4D antibody (10 μg/mL) for 24 h and centrifugation at 2000 × *g* for 5 min. The characterisation of BG was performed according to previous research [31]. The chemical structure and phase composition of BG were determined using FTIR (VERTEX 33 Bruker, Germany), XRD (D8 Discover, Bruker, Germany) and XPS (Thermo Scientific K‐Alpha, USA). The morphologies of BG and BG@ab were measured by TEM (Tecnai G2 F20 S‐TWIN, USA), and the chemical composition of BG@ab was detected by energy‐dispersive X‐ray (EDX) spectroscopy.

The synthesis of PLGA–PEG–PLGA (PPP) was performed according to previous protocols [32]. In brief, PEG1500 (5.02 g), GA (1.05 g) and LA (9.0 g) were added into a three‐neck flask at 150°C for 2 h, and Sn (Oct)2 (30 μL, 0.3%, w/w) was added under a nitrogenous atmosphere for 10 h. The mixed solution was added into deionised water at 4°C and stirred overnight. The PPP copolymer was obtained after freeze‐drying for 24 h, and the yield exceeded 80%. Finally, 3%–5% BG@ab was incorporated into a 20% solution of PPP at 4°C, stirred continuously for 2 h, and the gel (Gel‐BG@ab) formed at 37°C.

### 2.3 Induction of BRONJ Lesions in Mice

To establish the BRONJ models, TCRδ^−/−^ mice and WT mice were randomly divided into two groups, respectively: the untreated control group (18 mice) and the ZOL treated group (18 mice). The control group comprised mice exerting tooth extraction without ZOL injection. The ZOL treated group received intraperitoneal injection of ZOL (500 μg/kg) or physiological saline twice a week for 3 weeks before maxillary first molar extraction [33], and ZOL injection continued following extraction. The dose of ZOL was estimated using allometric scaling related to human tumour doses [34]. The tissues of the jawbone and gingiva were collected post‐extraction at 4 weeks, and BRONJ lesions were determined on the basis of (1) necrotic bone exposure, (2) abnormal epithelial proliferation and (3) epithelial fistula formation [35, 36]. Tissues from each group were separately sent for RNA sequencing (six mice), flow cytometry (six mice), Micro‐CT analysis, and histologic and immunohistochemical staining (six mice).

### 2.4 The Application of Gel‐BG@Ab in Rat BRONJ Models

To investigate the therapeutic efficacy of Gel‐BG@ab in treating BRONJ, we employed a rat BRONJ model. Rats were excluded due to the insufficient size of their extraction sockets, which could not accommodate hydrogel implantation and mucosa suture. Thus, 8‐week‐old female SD rats (40 rats) weighing 180–200 g were used for establishing a BRONJ model according to previously described methods [37]. The rats were randomly divided into three groups: the hydrogel untreated control group (No‐Tx, six rats), the hydrogel group (Gel‐BG, six rats) and the hydrogel combined with antibody group (Gel‐BG@ab, six rats). Equal amounts of composite hydrogel or saline were injected into the bone defects, and the wound was sutured with a small knot. The maxillary bone tissues of the rats were collected after treatment for 4 and 8 weeks. Tissues from each group were separately sent for Micro‐CT analysis and histologic and immunohistochemical staining.

### 2.5 Microcomputed Tomography (Micro‐CT)

Micro‐CT (Scanco μCT 40; Scanco Medical, Swiss) was used for scanning the maxillary bone of BRONJ lesions. Micro‐CT was performed at 55 kV and 145 μA with an integration time of 200 ms using a cylindrical tube. The resolution was 10 μm per pixel, and the local window size was (50 × 50 × 50 voxels). Three‐dimensional images of the maxilla were reconstructed using MicroView. The bone parameters, including the coronal bone mineral density (BMD, g/cm^3^) and the coronal and trabecular bone volume (BV, cm^3^), were calculated via a CT analyser (version 1.15.4.0, Belgium). The bone parameters, including the coronal BMD (g/cm^3^), the BV (mm^3^), total volume (TV, mm^3^) and the ratio of bone volume (BV/TV) were determined. The scanning and analysis of the data followed published guidelines [29, 38].

### 2.6 Histologic and Immunohistochemical Studies

The maxillary bones of the mice and rats were fixed, decalcified, paraffin‐embedded, sectioned (8 μm) and stained with H&E. Necrotic bone cells were defined as bone cell voids without cellular components or abnormally condensed small nuclei [36]. The tissue sections were subjected to Masson's trichrome, immunofluorescence and immunohistochemical staining. The first antibodies of anti‐osterix (1:200, GB111900‐100, Servicebio), anti‐osteocalcin (1:200, GB11233‐100, Servicebio), anti‐CD3 (1:100, sc‐20047, Santa Cruz), anti‐CD4 (1:100, sc‐19641, Santa Cruz), TCRγδ (1:100, 118108, BioLegend) and anti‐F4/80 (1:100, sc‐377009, Santa Cruz) were incubated overnight at 4°C, followed by IgG‐HRP or Alexa Fluor 488‐conjugated IgG incubation for 2 h at room temperature. The isotype‐matched control antibodies were used as the negative controls. Fluorescence images were captured under a microscope (Stellaris LAS X, Leica), and the percentage of positive cells per unit area was calculated by ImageJ software. The percentage of positive area was calculated as (positive area/total tissue area) × 100%. Positive cells were defined as regions with fluorescence intensity > 3× background standard deviation. All analyses were performed in a blinded manner by two independent operators.

### 2.7 RNA Sequencing

The RNA sequencing of the sockets of WT and TCRδ^−/−^ mice (treated with/without ZOL) was performed by Novogene Co. Ltd. (Beijing, China). The total RNA was extracted from the sockets using the TRIzol reagent following the manufacturer's protocol. RNA quality was assessed by Agilent 2100 and quantified using ND‐2000 (NanoDrop Technologies). The barcoded libraries for next‐generation sequencing were prepared using the TruSeqTM RNA Sample Prep Kit (Illumina, USA) according to the manufacturer's instructions, and the paired‐end sequencing (150 bp) was performed on an Illumina HiSeq 4000 platform. Differential analysis was conducted using the R software package (version 3.40.6) to identify differentially expressed genes between WT mice and TCRδ^−/−^ mice, and all genes with a fold change > 1.5 were identified as DEGs. Kyoto Encyclopedia of Genes and Genomes (KEGG) enrichment analyses were conducted, and a significant cut‐off point of *p* < 0.05 was used to determine significant enrichment. Gene set enrichment analysis (GSEA) was conducted using the GSEA software (version 3.0).

### 2.8 γδ T Cell Isolation and Flow Cytometry Analysis

To assess the proportion and number of γδ T cells in the sockets of WT and TCRδ^−/−^ mice 2 weeks after tooth extraction. The single cell suspension of mouse gingival tissues was obtained as previously described [39]. Briefly, the gingival tissues were cut into 1 mm pieces and placed in digestive buffer containing collagenase IV (2 mg/mL, Sigma Aldrich), collagenase II (1 mg/mL, Life Technologies) and DNase I (1 mg/mL, Roche). The tissues were incubated for 30 min at 37°C on a shaker at 150 rpm, and then mashed through a 70 μm cell strainer after digestion. The cell pellets were collected by centrifugation at 500 × *g* for 5 min. The dissociated cells were washed three times and the dead cells were excluded by Live/Dead fixable dye (565388, BD Biosciences). Staining for CD45 (30‐F11, BioLegend), CD3 (145‐2C11, BioLegend), γδ TCR (GL3, 118108, BioLegend) and Sema4D (147605, BioLegend) for 30 min on ice, with IgG used as an isotype control. Samples were acquired using a flow cytometer (Beckman, USA) and analysed with FlowJo software (BD Biosciences) [36, 40].

### 2.9 Expansion of γδ T Cells Ex Vivo

The spleens were harvested from WT and TCRδ^−/−^ mice, and the immune cells were obtained after tissue grinding and red cell deletion. CD3^+^ γδTCR^+^ or CD3^+^ T lymphocytes were sorted using a FACS Aria flow cytometer [41, 42]. The sorted T cells were cultured in 24‐well plates precoated with anti‐γδ TCR antibodies (10 μg/mL, 107502, BioLegend) and anti‐CD28 (1 μg/mL, 102116, BioLegend) overnight for activation. RPMI‐1640 medium supplemented with IL‐2 (20 ng/mL, 210‐21, PeproTech) and 10% FBS was used for culture. The activated cells were then transferred to new wells for expansion without anti‐γδ TCR and anti‐CD28. ZOL (0, 0.5, 5 and 15 μM) was added to the culture medium for 3–6 days. The proliferation, purity and Sema4D expression of γδ T cells after ZOL induction for 3 and 6 days was determined by flow cytometry. The supernatant of γδ T cells was collected after changing to fresh medium for 2 days of culture without ZOL. To measure the protease for Sema4D shedding, γδ T cells were pretreated with the metalloprotease inhibitors GM6001 (50 or 100 nM, MCE) and TAPI‐2 (10 or 20 nM, MCE) for 6 h before ZOL induction. Furthermore, γδ T cells were treated with MMP2 (1 μg/mL) and/or MMP9 (1 μg/mL) for 48 h in the absence or presence of metalloprotease inhibitors for 48 h, and sSema4D levels in the supernatants were determined by ELISA.

### 2.10 ALP Staining and Enzyme Activity Analysis

ALP staining kit (C3026, Beyotime) and enzyme activity assay kit (P0321, Beyotime) were used following the manufacturer's protocol. In brief, osteoblasts were cultured in a mixture of γδ T cell supernatant and osteogenic induction medium for 7 days, and the mixed medium was changed every 2 days. The osteogenic induction medium contained β‐glycerophosphate (G9422, Sigma), vitamin C (A4544, Sigma) and dexamethasone (D4902, Sigma). An anti‐Sema4D neutralising antibody (FMJ33910, Antibody System) and recombinant mouse Sema4D‐FC chimeric protein (5235‐S4B‐050, R&D Systems) were added coincidentally to the culture media with the osteogenic induction medium for different experiments; the final concentration was 100 ng/mL, respectively.

For ALP staining, the cells were fixed with 4% paraformaldehyde for 15 min and washed with PBS for three times; then stained with ALP staining solution for 45 min. The staining solution was rinsed with water and air dried.

For ALP activity measurement, cultured cells were collected in a lysis buffer on ice and subjected to ultrasonic disruption. The supernatant was collected after centrifugation at 10,000 × rpm for 15 min, and ALP activity and the total protein were measured according to the manufacturers' instructions. ALP activity should be normalised to total protein.

### 2.11 Molecular Interaction Simulations

The STRING database was used to search the interaction between Plexin‐B1/2 and proteins. Molecular docking was employed to analyse the interaction between Plexin‐B1/2 and mTOR [43]. Briefly, a specific mTOR protein (Q9JLN9) was identified from the UniProt database, and its protein structure was predicted using AlphaFold2. The protein crystal structure of Plexin‐B1 (8BB7) and Plexin‐B2 (5E6P) was obtained from the RCSB PDB database. The Lamarkian GA was used as the docking algorithm to select the conformation with the lowest docking energy as the primary conformation of protein. Subsequently, the separate docking of Plexin‐B1/2 with mTOR was performed using ZDock server, and the binding affinity between the proteins was evaluated by PRODIGY webserver. Molecular docking was accomplished using AutoDock Vina software after determining the Grid Box size and genetic algorithm. Finally, the visualisation and analysis of the bond interactions between the proteins were performed using Pymol software.

### 2.12 Quantitative PCR and Western Blot Analysis

The osteoblasts (MC3T3‐E1 cells) were cultured with the mix of γδ T cell supernatant and osteogenic induction medium. Effective *Plxnb1*, *Plxnb2* siRNA and control siRNA (Tsingke Biotech) were obtained from the supplier. When the cells reached 50% confluence, they were transfected with *Plxnb1*, *Plxnb2* siRNA using TSnanofect V2 Transfection Reagent (Tsingke Biotech) according to the manufacturer's protocol. The siRNA and qPCR primers used are listed in Table S1. Total RNA was extracted from cells using RNA extraction kits (AG21022, AG21023, Agbio), and RNA was reverse‐transcribed using kits (AG11707, Agbio). Next, qRT‐PCR was performed in three duplicates using a SYBR Green PCR Master Mix (AG11701, Agbio) and normalised to GAPDH. Each experiment was conducted independently at least three times.

The osteoblasts were lysed after culture or treatment. The proteins of osteoblasts were separated by SDS–PAGE and transferred to PVDF membranes. The membranes were incubated with primary antibodies against Sema4D (53108S, Cell Signaling Technology), Plexin‐B1 (sc‐28,372, Santa Cruz or 23,795–1‐AP, Proteintech), Plexin‐B2 (sc‐373969, Santa Cruz or 10602‐1‐AP, Proteintech), p‐mTOR (sc‐293133, Santa Cruz), mTOR (sc‐517464, Santa Cruz), ALP (DF6225, Affinity), OCN (DF12303, Affinity) and GAPDH (10494‐1‐AP, Proteintech). Subsequently, the membranes were incubated with horseradish peroxidase (HRP)‐conjugated secondary antibodies (1:4000, 7074, Cell Signaling Technology), and the protein bands were detected using enhanced chemiluminescence (ECL, SQ20, Epizyme, China) according to the manufacturer's instructions. The intensities of protein bands were quantified and normalised to GAPDH.

### 2.13 ELISA

To measure the content of sSema4D, MMP2, MMP3 and MMP9 in the tissues of extraction sockets and the supernatant of γδ T cells, an ELISA assay was performed. The kits of sSema4D (ELK5378, ELK Biotechnology), MMP2 (E‐EL‐M0780, Elabscience), MMP3 (E‐EL‐M0626) and MMP9 (E‐EL‐M3052) were used following the manufacturer's instructions. Briefly, the tissues were homogenised by a Tissue Homogeniser (Servicebio, Wuhan) to collect the supernatant. The supernatant of tissues or cells was added into the wells and incubated for 2 h at 37°C to allow antigen–antibody binding after 3% BSA Blocking for 1 h. The unbound molecules were washed with PBST, and the primary antibody was added and incubated for 1 h at room temperature; then the secondary antibody was incubated after washing. Finally, the substrate was added, and the absorbance was measured at 450 nm using an ELISA plate reader to quantify antigen concentration via a standard curve.

### 2.14 Live/Dead Staining and Cell Viability Analysis

After osteoblasts were cultured on gel PPP or Gel‐BG@ab (3% or 5% BG) for 48 h, AOPI dyes (Beyotime, China) were incubated with cells for 20 min at 37°C in the dark to measure live/dead cells. Images were taken with a Stellaris LAS X (Leica, Germany). Cell viability was assayed using a CCK‐8 assay kit according to the manufacturer's protocol. In brief, cells were seeded on the gel PPP or Gel‐BG@ab (3% or 5% BG) at 5000 cells/well in 96‐well plates (with 100 μL culture medium) and incubated for 24 h. Ten μL CCK‐8 reagent was added to incubate for 2 h; the absorbance was measured at 450 nm by Microplate Reader.

### 2.15 Statistical Analysis

All the data were obtained from at least three independent experiments and are shown as mean ± SD. Comparison between two groups was analysed using a two‐tailed unpaired Student's *t*‐test. Comparison between multiple groups with one variable was analysed using one‐way analysis of variance (ANOVA) with Dunnett's test or Tukey's multiple‐comparison test. *P* values less than 0.05 were considered to indicate statistical significance. Statistical analyses and graphical representation of data were carried out with GraphPad Prism 7.0.

## 3 Results

### 3.1 Increased γδ T Cell Infiltration in the Lesions of BRONJ

**FIGURE 1:**
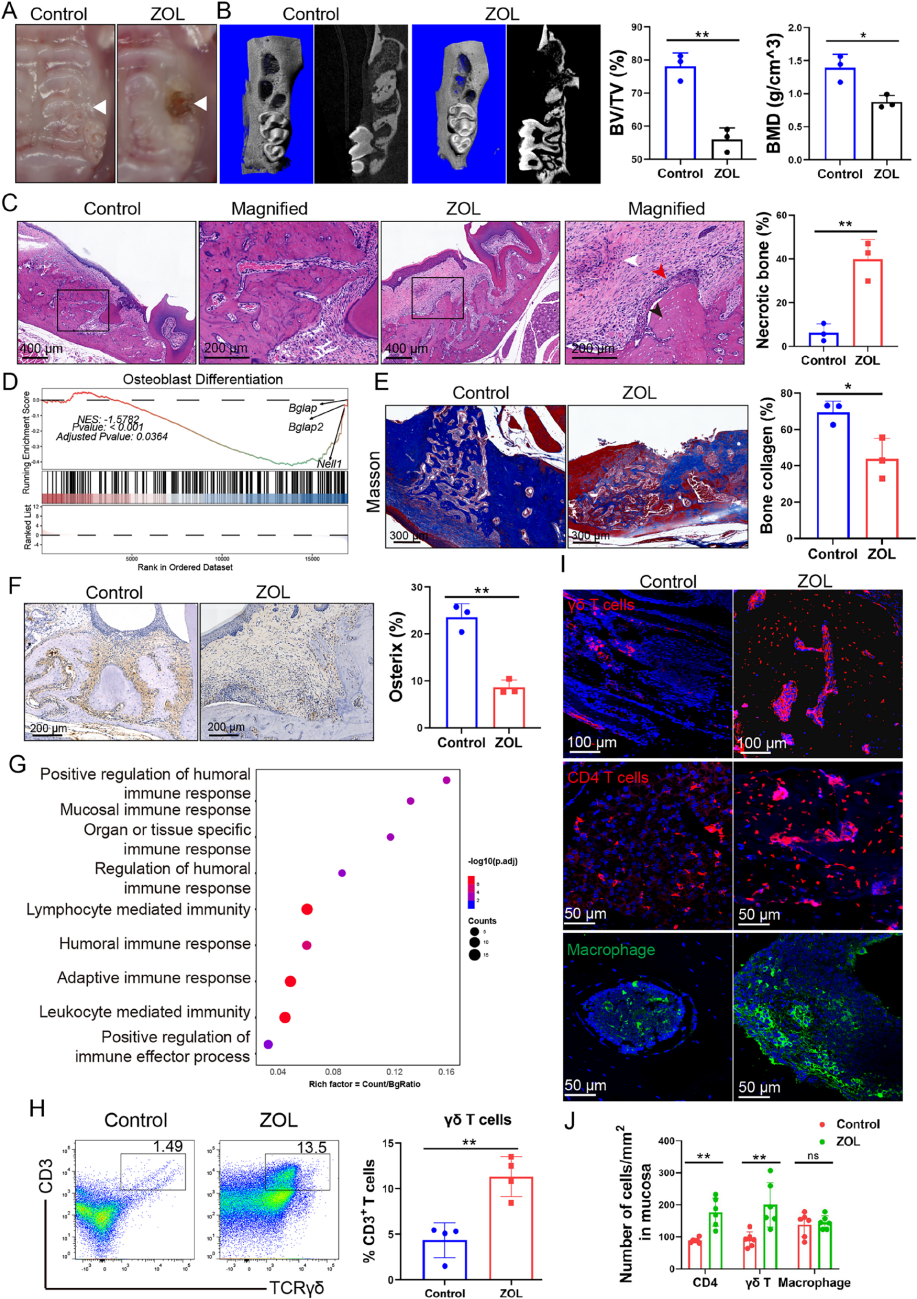
Increased γδ T cell infiltration in nonhealing BRONJ lesions. (A) Representative images of the extracted sockets in control and ZOL‐treated mice at 4 weeks. Gingival mucosa swelling was observed as indicated by white arrows in ZOL‐treated mice. (B) Representative micro‐CT images of the sockets of control and ZOL‐treated mice, and the relative bone volume (BV/TV) and bone mineral density (BMD) were significantly decreased in ZOL‐treated mice. *n* = 3, **p < 0.01, *p < 0.05. (C) H&E staining of sockets showed necrotic bone and infiltrating immune cells, and the ratio of necrotic bone in the sockets was measured. White arrows in ZOL‐treated mice indicated infiltration of inflammatory cells, red arrows indicated abnormal epithelial proliferation, and black arrows indicated dead bone cells with vacuolated nuclei. Scale bars, 200 and 400 μm. *n* = 3, **p < 0.01. (D) GSEA results showed the osteoblast differentiation pathway was significantly down‐regulated in ZOL‐treated mice. Adjusted *p* < 0.05. (E) Masson's trichrome stained image of sockets showed new bone collagen (blue) decreased in ZOL‐treated mice, and the mature bone tissue or fibrous collagen (red) was much more than that in control mice. Scale bars, 300 μm. *n* = 3, *p < 0.05. (F) IHC staining image showed that the osterix positive cells in extraction sockets decreased in ZOL‐treated mice. Scale bars, 200 μm. *n* = 3, **p < 0.01. (G) KEGG analysis of DEGs in control and ZOL‐treated mice was mainly enriched in pathways related to immune response. (H) Representative scatter plots of γδ T cells in the sockets were presented, and the percentages of γδ T cells in ZOL‐treated mice were much more than that in control mice. *n* = 4, **p < 0.01. (I) Immunofluorescence staining of CD4^+^ T cells (red), γδ T cells (red) and macrophages (green) in the sockets was presented. Scale bars, 50 and 100 μm. (J) The number of CD4^+^ T cells and γδ T cells increased in ZOL‐treated mice, and macrophages did not change much in the sockets. *n* = 6, **p < 0.01, ns indicates no significance. Data are mean ± SD.

To explore whether the local immune disorder and bone turnover dysfunction contribute to the pathogenesis of BRONJ, the mice BRONJ model was established, referred to in other studies [28]. The BRONJ lesions were identified based on necrotic bone exposure, abnormal epithelial proliferation and epithelial fistula formation [35, 36]. ZOL‐injected mice exhibited local swelling and open alveolar sockets (Figure 1A), reduced new bone formation (Figure 1B), apparent bone necrosis, increased inflammatory cell infiltration and abnormal epithelial overgrowth (Figure 1C). GSEA analysis indicated that osteoblast differentiation was significantly downregulated in the ZOL treated group (Figure 1D). The amount of blue‐stained newly formed bone collagen (Figure 1E) and osterix‐positive cells were noticeably decreased in the BRONJ lesions (Figure 1F). It suggested a marked inhibition of osteoblast differentiation and new bone formation in BRONJ lesions.

It has been reported that the disruption of immune balance promotes BRONJ [11, 21, 44]. KEGG enrichment analysis revealed that DEGs of the tissues from the extracted sockets were mainly enriched in pathways related to mucosal immune response (Figure 1G). γδ T cells were reported to have played vital roles in immune surveillance and bone metabolism [14, 40], and they could be effectively activated by ZOL [23, 24, 25]. Thus, we tried to figure out whether ZOL influences the population and function in BRONJ. γδ T cells were identified as CD3^+^ TCRγδ^+^ T cells demonstrated by flow cytometry in mucosal tissues; γδ T cells were 2.6 times more abundant in BRONJ lesions than in the control (11.32% vs. 4.34%) (Figure 1H). IF staining and cytometry analysis revealed that γδ T cells were significantly higher in BRONJ lesions than in control (Figure 1I,J). In all, these results showed the inhibition of osteoblast differentiation and the increase of γδ T cell infiltration in BRONJ.

### 3.2 γδ T Cells Increased the Incidence of BRONJ Lesions by Inhibiting the Osteoblast Differentiation

**FIGURE 2:**
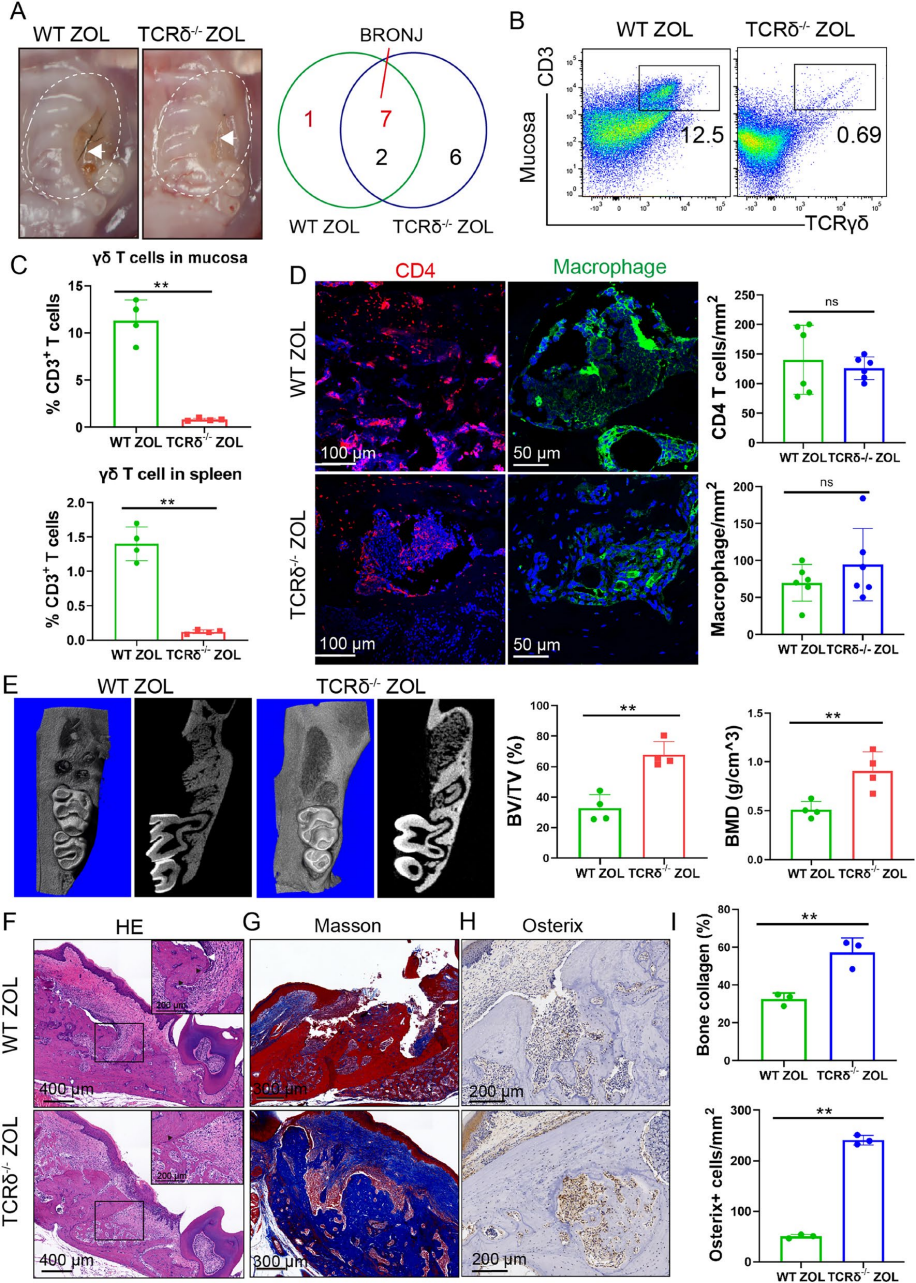
Osteogenic differentiation inhibition and BRONJ incidence diminished in γδ T cell‐knockout mice. (A) Representative images showed the extracted sockets in control and WT ZOL and TCRδ^−/−^ ZOL mice at 4 weeks, and the white dashed lines indicate the extent of mucosal swelling. The number of WT ZOL and TCRδ^−/−^ ZOL mice suffering from BRONJ was analysed. *n* = 8. (B) Representative scatter plots showed the percentage of γδ T cells in the extraction sockets and spleen in WT ZOL and TCRδ^−/−^ ZOL mice. (C) The γδ T cell percentage in the extraction sockets was measured, and ZOL induced γδ T cell expansion in WT mice but not in TCRδ^−/−^ mice. *n* = 4, **p < 0.01. (D) Immunofluorescence images showed CD4^+^ T cells (red) and macrophages (green) in the sockets, and the number of CD4^+^ T cells and macrophages did not change in TCRδ^−/−^ mice. Scale bars, 50 and 100 μm. *n* = 6, ns indicates no significance. (E) Micro‐CT images were presented, and the results of BV/TV and BMD showed that the deletion of γδ T cells increased the new bone formation in TCRδ^−/−^ ZOL mice. *n* = 4, **p < 0.01. (F) H&E staining of extraction sockets in WT ZOL and TCRδ^−/−^ ZOL mice, and the vacuolated nuclei and abnormal epithelial cells were much less in TCRδ^−/−^ ZOL mice than in WT ZOL mice. Scale bars, 200 and 400 μm. (G) Masson's trichrome staining of extraction sockets showed that the new bone collagen (blue) increased in TCRδ^−/−^ ZOL mice compared to WT ZOL mice. Scale bars, 300 μm. (H) IHC staining showed that the osterix positive cells in the sockets of TCRδ^−/−^ ZOL mice were much more than in WT ZOL mice. Scale bars, 200 μm. (I) The percentage of collagen deposition and the number of osterix‐positive cells were analysed in the sockets. *n* = 3, **p < 0.01. Data are mean ± SD.

It has been reported that the prolonged residency and activation of γδ T cells might be important factors contributing to abnormal epithelial proliferation in BRONJ [21]. To investigate the potential role of γδ T cells in the pathological development of BRONJ and whether the immune dysregulation of γδ T cells inhibited osteoblast differentiation, γδ T cell‐deficient (TCRδ^−/−^) mice were used. The gingiva wound healed well in TCRδ^−/−^ ZOL mice compared to the pit‐shaped wounds of WT ZOL mice, and the incidence of ZOL‐induced BRONJ lesions in TCRδ ZOL mice was significantly lower TCRδ^−/−^ ZOL mice (25% vs. 87.5%) (Figure 2A). γδ T cells were absent in mucosal tissues, blood and spleens in TCRδ^−/−^ mice, which accounted for less than 0.5% of the total T cells (Figure S1A–C), and γδ T cells did not increase after ZOL administration and tooth extraction (Figure 2B,C). Immunofluorescence staining also revealed that the quantity and staining area of CD4^+^ T cells and macrophages did not change (Figure 2D), which indicated that γδ T cell absence of γδ T cells had little effect on CD4^+^ T cells and macrophages. Furthermore, micro‐CT results showed that the bone volume fraction (BV/TV) and BMD were significantly greater at the sockets in TCRδ^−/−^ ZOL mice than in WT ZOL mice (Figure 2E). The abnormal epithelial overgrowth and necrotic bones in TCRδ^−/−^ ZOL mice were much less than in WT ZOL mice (Figure 2F and Figure S1D). Blue‐stained collagen deposition and osterix‐positive cells were much more abundant in the sockets of TCRδ^−/−^ ZOL mice than in WT ZOL mice (Figure 2G–I). In all, these findings suggested that γδ T cells inhibited collagen generation and osteoblast differentiation in BRONJ lesions.

### 3.3 Sema4D Derived From γδ T Cells Was the Vital Molecule Inhibiting the Osteoblast Differentiation in BRONJ Lesions

**FIGURE 3:**
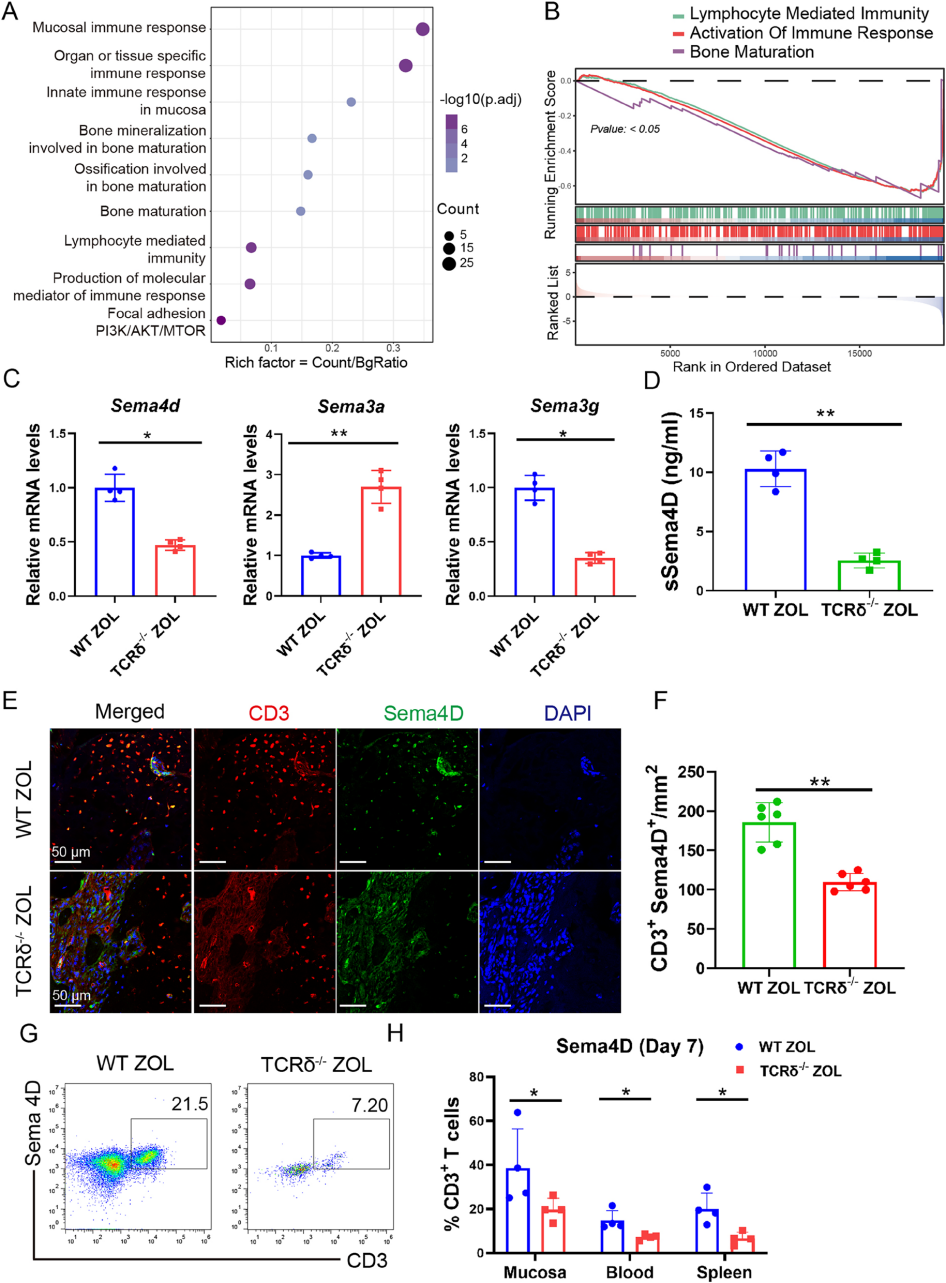
Sema4D derived from γδ T cells. (A) KEGG analysis of the DEGs of WT ZOL and TCRδ^−/−^ ZOL mice. (B) GSEA results of WT ZOL and TCRδ^−/−^ ZOL mice showed lymphocyte‐mediated immunity, activation of immune response and bone maturation pathways. (C) The mRNA levels of *Sema4d* and *Sema3g* decreased, and *Sema3a* increased in the sockets of TCRδ^−/−^ ZOL mice compared to WT ZOL mice. *n* = 4, *p < 0.05, **p < 0.01. (D) The level of sSema4D in the sockets was detected by ELISA. *n* = 4, **p < 0.01. (E) Colocalization staining of CD3 and Sema4D in the sockets at 7 days after tooth extraction in WT ZOL and TCRδ^−/−^ ZOL mice. Scale bars, 50 μm. (F) The number of CD3^+^ Sema4D^+^ T cells in the sockets of TCRδ^−/−^ ZOL mice was much less than that in WT ZOL mice. **p < 0.01. (G) Representative scatter plots of CD3^+^ Sema4D^+^ T cells were shown. (H) The Sema4D expression on CD3^+^ T cells of mucosa, blood and spleen in WT ZOL and TCRδ^−/−^ ZOL mice was analysed on day 7. *n* = 4, *p < 0.05. Data are mean ± SD.

To further investigate how γδ T cells impacted on osteoblast function, the analysis of DEGs of WT ZOL and TCRδ^−/−^ ZOL mice was performed. KEGG and GSEA analysis showed that DEGs of WT ZOL and TCRδ^−/−^ ZOL mice were mostly enriched in mucosal immune response, lymphocyte‐mediated immunity, bone maturation and mTOR‐related pathways (Figure 3A,B). We examined the genes in the four pathways, and semaphorins showed significant differences. The mRNA level of *Sema3g* and *Sema4d* was decreased in TCRδ^−/−^ ZOL mice and *Sema3a* was increased in TCRδ^−/−^ ZOL mice compared to that of WT ZOL mice, while *Sema3e* and *Sema4a* were too low to be detected in both groups (Figure 3C). Based on the above results, *Sema4a* and *Sema3g*, which were mainly secreted by endothelial cells [45] were excluded; we only focused on Sema4D of γδ T cells in BRONJ lesions.

The Sema4D contained membrane‐bound (mSema4D) and soluble forms (sSema4D) [46, 47]. To measure the mSema4D and sSema4D in BRONJ lesions, the flow cytometry and ELISA were performed, respectively. The sSema4D was much lower in the extraction sockets of TCRδ^−/−^ ZOL mice than that in WT ZOL mice measured by ELISA (Figure 3D). Immunofluorescence staining showed that the mSema4D on CD3^+^ T cells was significantly lower in TCRδ^−/−^ ZOL mice than that in WT ZOL mice (Figure 3E,F). Moreover, the proportion of Sema4D^+^ CD3^+^ T cells in the mucosa, blood, and spleens of TCRδ^−/−^ ZOL mice was much less than that in WT ZOL mice at 7 days after tooth extraction (Figure 3G,H). The reduction of sSema4D and mSema4D^+^ CD3^+^ T cells in TCRδ^−/−^ ZOL mice indicated that γδ T cells and their expression of Sema4D constitute an important role in BRONJ lesions.

### 3.4 Soluble Sema4D of Activated γδ T Cells Exerted Osteoblast Differentiation Inhibition In Vitro

**FIGURE 4:**
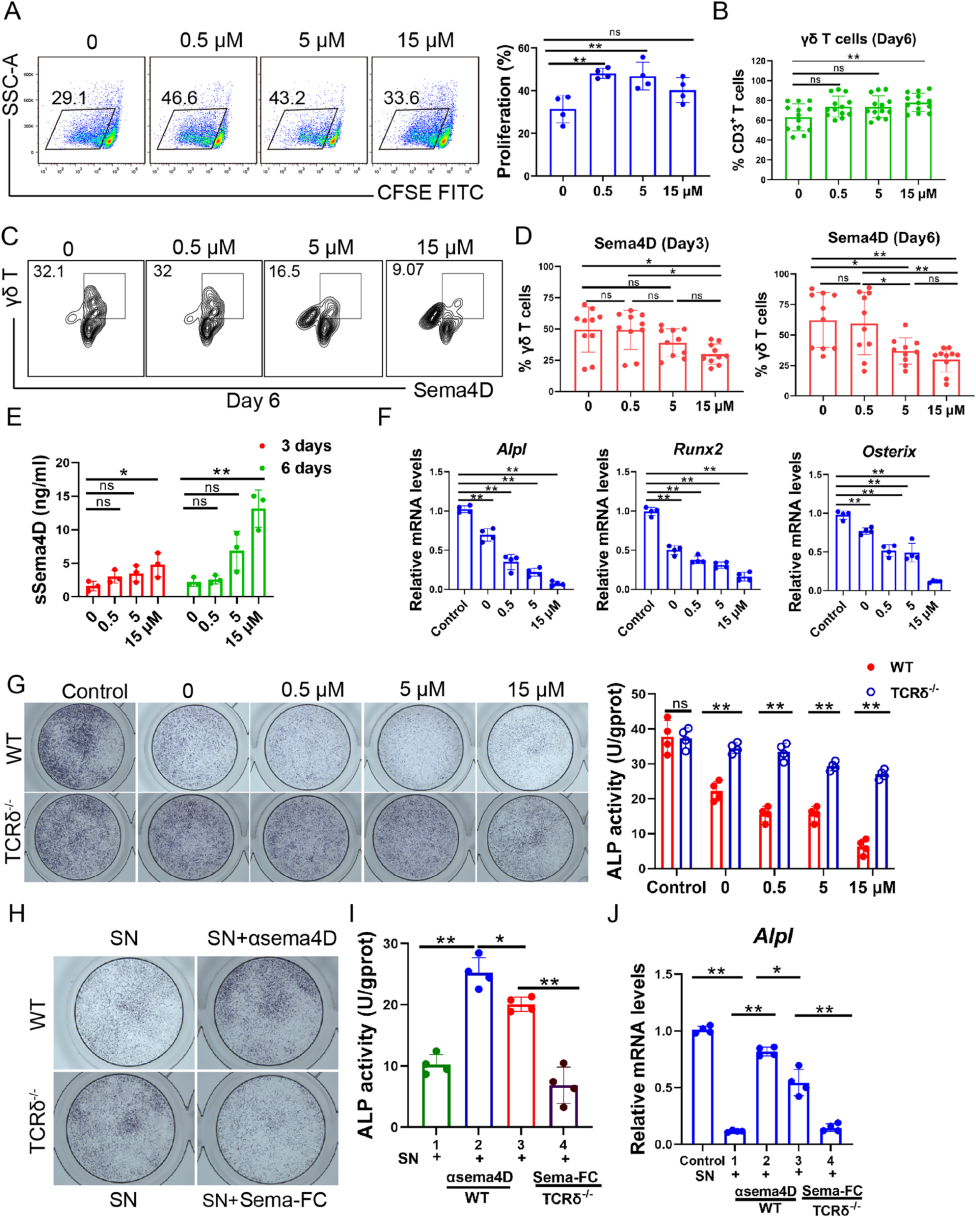
Soluble Sema4D of activated γδ T cells inhibited osteoblast differentiation. (A) Representative scatter plots of γδ T cell proliferation were shown, and ZOL increased the proliferation of γδ T cells from the spleens at different concentrations for 3 days. *n* = 4, **p < 0.01, ns indicates no significance. (B) Purity of γδ T cells incubated with ZOL for 6 days. *n* = 13, **p < 0.01, ns indicates no significance. (C) Representative contour plots of Sema4D^+^ γδ T cells. (D) The Sema4D expression on expanded γδ T cells decreased significantly at 15 μM ZOL treatment on day 3 and day 6. *n* = 10, *p < 0.05, **p < 0.01, ns indicates no significance. (E) The sSema4D in γδ T cell supernatant increased significantly at 15 μM ZOL treatment on day 3 and day 6. *n* = 3, *p < 0.05, **p < 0.01, ns indicates no significance. (F) The mRNA level of *Alpl*, *Runx2* and *Osterix* was measured after being cultured with γδ T cell supernatant from WT ZOL mice, and the mRNA levels declined in a dose‐dependent manner. *n* = 4, **p < 0.01. (G) ALP staining of osteoblasts after incubation with the γδ T cell supernatant for 7 days, and the supernatant of γδ T cells was collected from WT ZOL and TCRδ^−/−^ ZOL mice treated with different concentrations of ZOL. ALP enzyme activity was also analysed on day 7 after incubation with γδ T cell supernatant. The supernatant from WT mice showed much stronger inhibition of osteoblast differentiation than that of TCRδ^−/−^ mice. *n* = 4, **p < 0.01. ns indicates no significance. (H and I) The γδ T cell supernatant supplemented with anti‐Sema4D antibody (αSema4D) alleviated the inhibition of osteoblast differentiation; however, the Sema‐FC enhanced differentiation inhibition of the supernatant of TCRδ^−/−^ mice. SN, supernatant of γδ T cells. *n* = 4, *p < 0.05, **p < 0.01. (J) The mRNA level of *Alp* was measured after incubation with the γδ T cell supernatant supplemented with anti‐Sema4D antibody (αSema4D) and/or Sema‐FC. *n* = 4, *p < 0.05, **p < 0.01. Data are mean ± SD.

To confirm whether the level of Sema4D increased in activated γδ T cells, we cultured γδ T cells isolated from mouse spleens using anti‐TCRδ antibodies [41, 42], with the addition of ZOL (0, 0.5, 5 and 15 μM) [36]. ZOL increased the proliferation rate of γδ T cells (Figure 4A) and improved the purity of γδ T cells to 77.93% compared to anti‐TCRδ antibodies alone (69.23%) at day 6 (Figure 4B and Figure S2A). The expression of mSema4D on γδ T cells decreased at day 3 and day 6 (Figure 4C,D and Figure S2B); however, the sSema4D in the culture medium increased with rising concentrations of ZOL (Figure 4E). The results suggested that ZOL enhanced the proliferation and purity of γδ T cells but decreased Sema4D expression on activated γδ T cells in a dose‐dependent manner.

To clarify whether sSema4D produced by γδ T cells affects the differentiation of osteoblasts, the mRNA level and ALP staining were measured. We found that the γδ T cell supernatant from WT ZOL mice decreased the mRNA level of *Alpl*, *Runx2* and *Osterix* more significantly than that from TCRδ^−/−^ ZOL mice (Figure 4F). Moreover, the ALP staining and enzyme activity were significantly weaker in osteoblasts cultured with the cell supernatant from WT ZOL mice than that from TCRδ^−/−^ ZOL mice (Figure 4G). Sema4D neutralising antibody (αSema4D) could alleviate the inhibition of ALP caused by γδ T cell supernatant from WT ZOL mice, while Sema4D fragment (Sema‐FC) exacerbated the inhibition of ALP by supernatant from TCRδ^−/−^ ZOL mice (Figure 4H–J). Additionally, the αSema4D and Sema‐FC did not affect the proliferation and percentage of γδ T cells (Figure S2C,D). It suggested that sSema4D from γδ T cells exerts a major function inhibiting osteoblast differentiation.

### 3.5 sSema4D Inhibited Osteoblast Differentiation via Plexin‐B/mTOR Signalling

**FIGURE 5:**
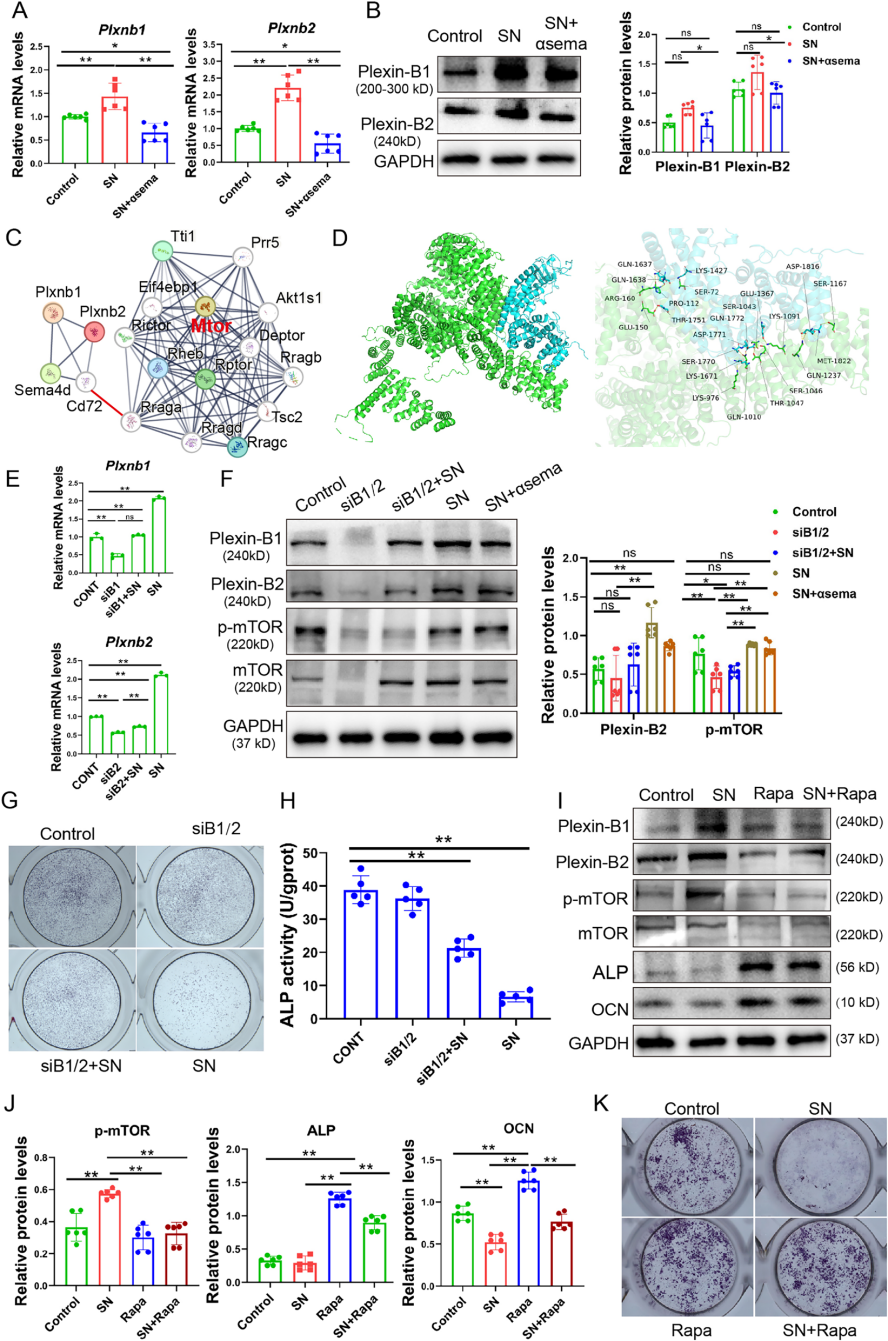
Sema4D inhibited osteoblast differentiation via Plexin‐B/mTOR signalling. (A) The mRNA levels of *Plxnb1* and *Plxnb2* in osteoblasts increased after SN incubation, and the anti‐Sema4D antibody inhibited the expression. SN, supernatant of γδ T cells. *n* = 6, *p < 0.05, **p < 0.01. (B) The protein levels of Plexin‐B1 and Plexin‐B2 in osteoblasts were measured, similar to the mRNA expression trend. *n* = 6, *p < 0.05; ns indicates no significance. (C) The STRING database showed that mTOR might be associated with Plexin‐B1 and Plexin‐B2. (D) The binding form and binding sites of mTOR and Plexin‐B2 were visualised by PyMOL. mTOR protein (green), Plexin‐B2 protein (blue). (E) The siRNA of *Plxnb1/2* inhibited the mRNA levels of *Plxnb1* and *Plxnb2* in osteoblasts treated with and/or γδ T cell supernatant. siB1 indicates the siRNA of *Plxnb1*, and siB2 indicates the siRNA of *Plxnb2*. *n* = 3, **p < 0.01; ns indicates no significance. (F) WB analysis showed that the siRNA of *Plxnb1/2* downregulated the protein level of Plexin‐B1, Plexin‐B2 and p‐mTOR in osteoblasts. *n* = 6, *p < 0.05, **p < 0.01; ns indicates no significance. (G) ALP staining of osteoblasts after incubation with siRNA, αSema4D or γδ T cell supernatant for 7 days, and the siRNA alleviated the inhibition of SN. (H) Analysis of ALP enzyme activity in osteoblasts after incubation with siRNA, αSema4D or γδ T cell supernatant for 7 days. *n* = 5, **p < 0.01. (I and J) WB showed the protein levels of Plexin‐B1, Plexin‐B2, p‐mTOR, mTOR, ALP and OCN after treatment with rapamycin and/or the supernatant. The rapamycin inhibited the expression of p‐mTOR and mTOR and cut off the pathway activated by the supernatant. *n* = 6, **p < 0.01. (K) ALP staining of osteoblasts after treatment with rapamycin and/or the supernatant for 7 days. Data are mean ± SD.

We needed to examine how sSema4D from γδ T cells inhibited osteoblast differentiation. The research has shown that Sema4D worked by binding to receptors such as Plexin‐B1, Plexin‐B2 and CD72 on target cells [46, 48]. Our results revealed that the supernatant of activated γδ T cells could upregulate the mRNA levels of *Plxnb1* and *Plxnb2* (Figure 5A) but not influence *Cd72* (Figure S3A). Western blot (WB) results showed a consistent trend (Figure 5B). We screened numerous proteins that interact with Plexin‐B1 and Plexin‐B2 using the STRING database and found that the mammalian target of rapamycin (mTOR) might be associated with Plexin‐B1/2 (Figure 5C). Since the mTOR signalling pathway was enriched in bulk‐seq results, as shown in Figure 3A, we further studied the interaction of mTOR and Plexin‐B1/2. The binding form and sites of mTOR and Plexin‐B1/2 were visualised by PyMOL, and the binding affinity between the proteins was tight (−16.1 kcal/mol) (Figure 5D, Figure S3B,C). The binding affinity served as an indicator of their binding capacity. A lower binding affinity signifies more stable ligand‐receptor binding. The binding energy is typically below −5 kcal/mol, indicating a solid binding force [49]. These results suggested that Sema4D might activate the mTOR signalling pathway to inhibit osteoblast differentiation.

To investigate the role of Sema4D on the mTOR pathway, the siRNA of *Plxnb1/2* was used to knock down *Plxnb1/2* expression (Figure 5E), and the protein levels of p‐mTOR and mTOR significantly decreased following *Plxnb1/2* knockdown (Figure 5F). ALP staining and enzyme activity assays revealed that *Plxnb1/2* knockdown alleviated the inhibitory effect of the γδ T cell supernatant on osteoblast differentiation (Figure 5G,H). Furthermore, rapamycin (mTOR inhibitor) inhibited the protein level of p‐mTOR and mTOR induced by the γδ T cell supernatant, increased ALP and osteocalcin (OCN) expression, without affecting the Plexin‐B1/2 levels (Figure 5I,J and Figure S3D). Rapamycin alleviated the inhibitory effect of the γδ T cell supernatant on osteoblast differentiation (Figure 5K). These results suggested that sSema4D inhibited osteoblast differentiation through the Plexin‐B1/2‐mTOR pathway.

### 3.6 Autocrine MMP3 Secretion Induced Membrane Bound Sema4D (mSema4D) Cleavage on γδ T Cells

**FIGURE 6:**
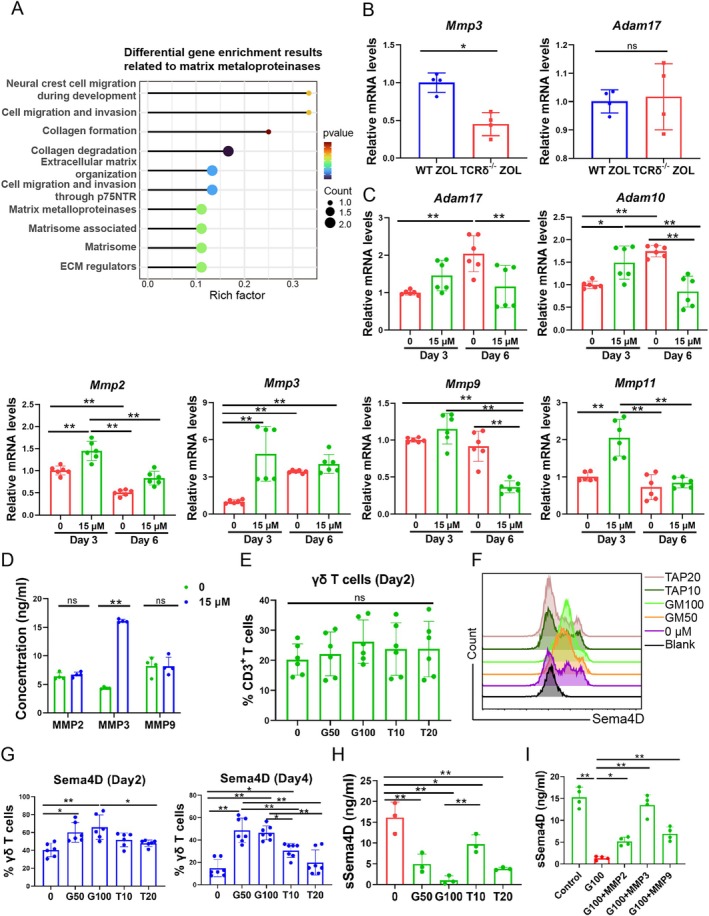
Autocrine MMP3 secretion induced mSema4D cleavage in γδ T cells. (A) KEGG analysis of DEGs in WT ZOL and TCRδ^−/−^ ZOL mice was highly enriched in matrix metalloproteinase (MMP)‐related pathways. **(B)** The mRNA levels of *Mmp3* and *Adam17* in the extraction sockets of WT ZOL and TCRδ^−/−^ ZOL mice. *n* = 4, *p < 0.05, ns indicates no significance. (C) The mRNA levels of *Adam17*, *Adam10*, *Mmp2*, *Mmp3*, *Mmp9* and *Mmp11* after γδ T cells were incubated with 15 μM ZOL for 3 and 6 days. Only the mRNA level of *Mmp3* increased significantly and kept a high level after 6 days incubation. *n* = 6, *p < 0.05, **p < 0.01. (D) The protein level of MMP3 was much higher than MMP2 and MMP9 in the γδ T cell supernatant measured by ELISA. *n* = 4, **p < 0.01, ns indicates no significance. (E) The proportions of γδ T cells after treatment with GM6001 and TAPI‐2. GM6001 and TAPI‐2 are MMP inhibitors. T10 and T20 indicate 10 and 20 nM TAPI‐2, respectively; G50 and G100 indicate 50 and 100 nM GM6001, respectively. *n* = 6, ns indicates no significance. (F) The expression of Sema4D on γδ T cells after treatment with GM6001 and TAPI‐2. (G) Analysis of Sema4D expression in γδ T cells after treatment with GM6001 or TAPI‐2 on day 2 and 4. *n* = 6, *p < 0.05, **p < 0.01. (H) The level of sSema4D in the supernatant of γδ T cells after treatment with GM6001 or TAPI‐2. *n* = 3, *p < 0.05, **p < 0.01. (I) The level of sSema4D in the supernatant of γδ T cells after incubating with MMP2, MMP3 and MMP9 in synergy with GM6001. *n* = 4, *p < 0.05, **p < 0.01. Data are mean ± SD.

How mSema4D shed from the γδ T cell membrane after ZOL activation has yet to be elucidated. Previous studies had reported that the enzymes of hydrolysis and cleavage were necessary for protein shedding from cytomembranes, and the metalloproteinases were reported as the most related enzymes [46, 50]. To figure out which metalloproteinases exert function in BRONJ, KEGG analysis was performed. DEGs of WT ZOL and TCRδ^−/−^ ZOL mice were enriched in biological processes related to matrix metalloproteinases (Figure 6A). We screened various metalloproteinases and only *Mmp3* and *Adam17* were detectable in BRONJ lesions (Figure 6B). Only *Mmp3* maintained a high level in γδ T cells during long‐term activation by ZOL (Figure 6C). Moreover, MMP3 significantly increased in the supernatant for 15 μM ZOL activation on day 6 (Figure 6D). These results showed that MMP3 might play a crucial role in the shedding of mSema4D.

To confirm whether the MMP3 was the key metalloprotease in the shedding mSema4D, we pretreated γδ T cells with metalloprotease inhibitors; GM6001 (ilomastat) predominantly inhibited MMPs [51], and TAPI‐2 primarily inhibited the expression of both MMPs and ADAMs [52, 53]. The proportion of γδ T cells was not influenced by the inhibitors (Figure 6E). The inhibitors significantly inhibited mSema4D shedding from γδ T cells on days 2 and 4 (Figure 6F,G) and decreased the content of sSema4D in the supernatant of γδ T cells (Figure 6H). The addition of recombinant MMP3 significantly increased the concentration of sSema4D in the supernatant, which exerted an antagonistic effect on the metalloprotease inhibitor (Figure 6I). These results suggested that ZOL promoted MMP3 activation and accelerated mSema4D shedding.

### 3.7 The Synthesis and Characteristics of the Composite Hydrogel (Gel‐BG@Ab)

**FIGURE 7:**
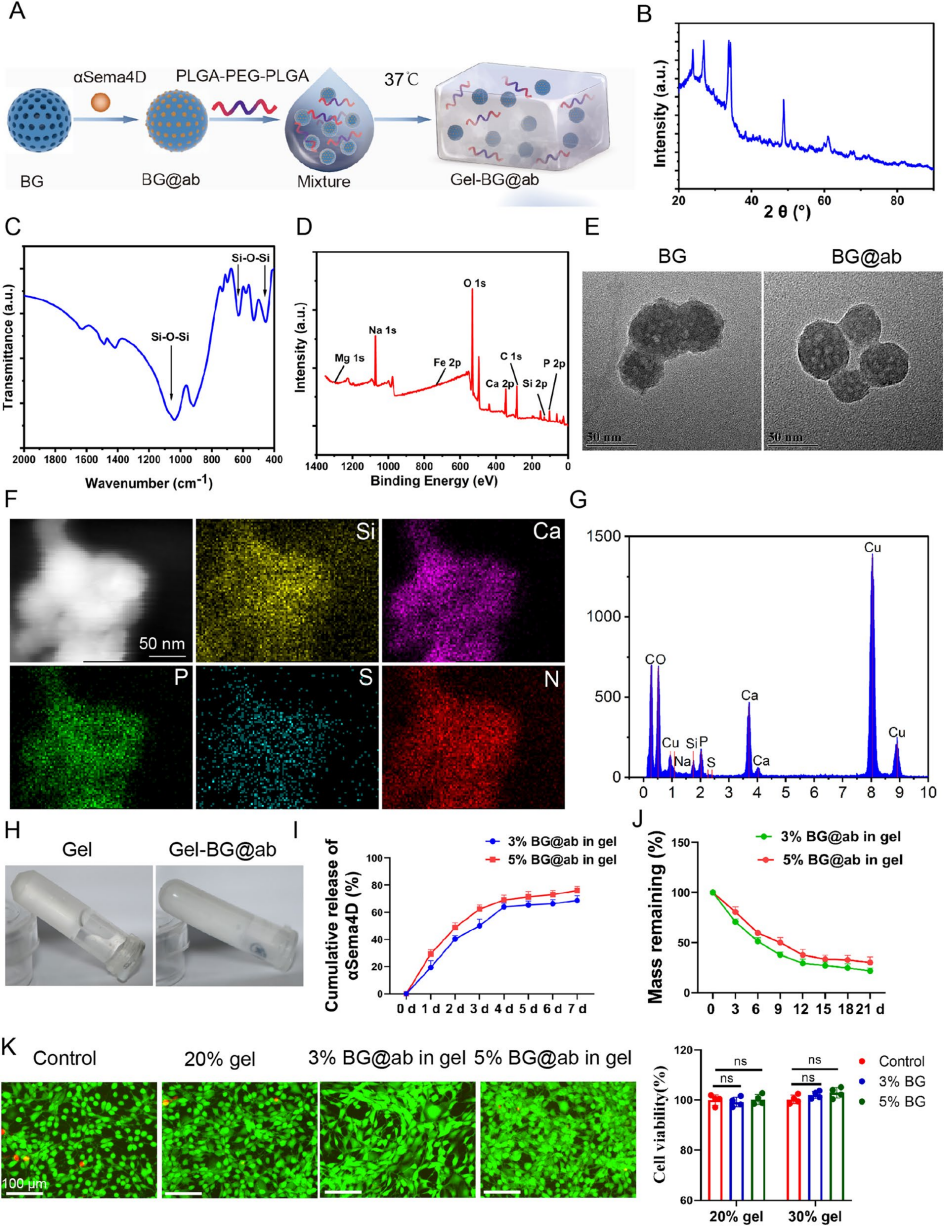
Gel‐BG@ab enabled sustained release of anti‐Sema4D. (A) Schematic illustration of the procedure of Gel‐BG@ab synthesis. BG, mesoporous bioactive glass; BG@ab, BG loaded with anti‐sema4D antibody; mixture indicates BG@ab added to a solution of PPP. (B) XRD pattern of BG. (C) FTIR spectra of BG. (D) XPS spectra of BG. (E) TEM images of BG and BG@ab. Scale bars, 50 nm. (F) TEM images of BG@ab and the corresponding element maps (Si, Ca, P, S and N). Scale bars, 50 nm. (G) EDX spectrum of BG@ab. (H) Images of the gel (PPP) and Gel‐BG@ab at 37°C. (I) The cumulative anti‐sema4D antibody released from the 3% BG and 5% BG in the hydrogel from day 0 to day 7. (J) The hydrogel contained BG@ab (3% or 5%) degraded gradually in PBS from day 0 to day 21. (K) AOPI staining images of osteoblasts cultured on the gel. 20% gel indicated 20% PPP, and 3% BG@ab and 5% BG@ab indicated the weight ratio of BG in the gel. Scale bars, 100 μm. The viability of osteoblasts cultured on the gel was measured via CCK‐8 assay kits. ns indicates no significance. Data are mean ± SD.

We investigated whether anti‐Sema4D antibodies would prevent or ameliorate BRONJ; we prepared the composite hydrogel of Gel‐BG@ab with local sustained release of anti‐Sema4D antibodies. The entire synthesis process of synthesising Gel‐BG@ab was illustrated in a schematic diagram (Figure 7A). X‐ray diffraction (XRD) analysis showed a broad diffuse diffraction peak around 30°, indicating the characteristic noncrystalline structural features of the mBG nanoparticles (Figure 7B). Fourier transform infrared (FTIR) spectroscopy detected two wide vibrational bands around 400–600 cm^−1^ and 900–1100 cm^−1^, which could be attributed to bending vibration and asymmetric stretching vibration of Si—O—Si, respectively (Figure 7C). X‐ray photoelectron spectroscopy (XPS) revealed that Si, Ca, P, C, Fe, Mg and Na were present in the BG nanoparticles (Figure 7D). Transmission electron microscopy (TEM) images showed the nanosized spherical feature of BG particles with a porous structure of approximately 1–5 nm (Figure 7E). The corresponding elemental content map revealed that BG@ab contained Si, Ca, P, S and N; S and N were the contents of anti‐Sema4D antibody (Figure 7F,G). It indicated successful loading of anti‐Sema4D antibody onto BG nanoparticles.

PPP is an FDA‐approved excipient widely used in disease treatment and tissue engineering [54, 55]. BG were uniformly dispersed in a PPP solution and formed hydrogel at 37°C (Figure 7H). The cumulative anti‐Sema4D antibody could release from the 3% BG and 5% BG in the hydrogel gradually (Figure 7I), and the hydrogel degraded gradually in PBS (Figure 7J). Neither the hydrogel nor the BG nanoparticles adversely affected the viability of osteoblasts (Figure 7K). These results demonstrated that Gel‐BG@ab could load and release anti‐Sema4D antibodies without affecting osteoblasts viability.

### 3.8 The Sustained Release of Sema4D by Gel‐BG@Ab Reduced the Formation of BRONJ Lesions

**FIGURE 8:**
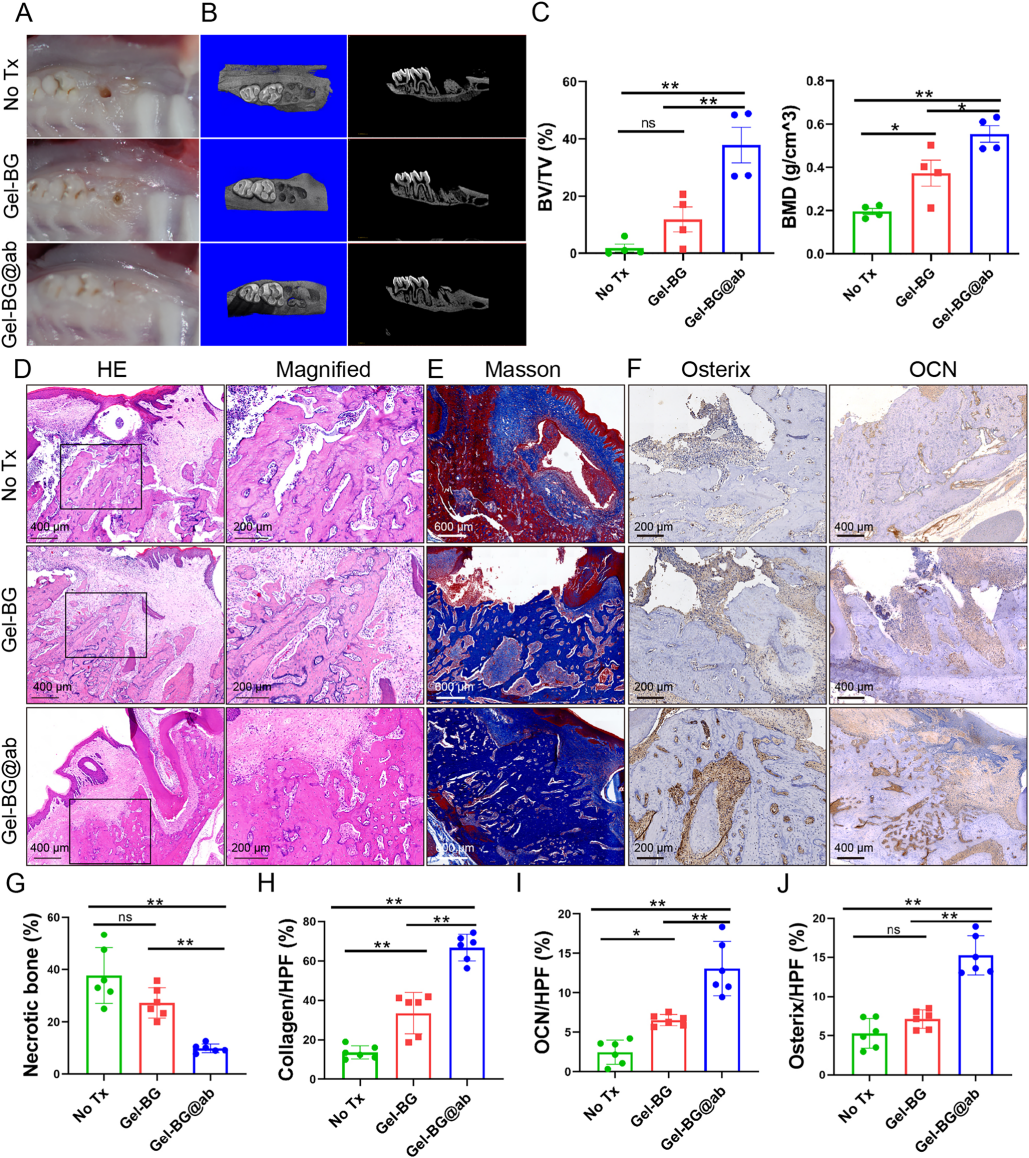
Gel‐BG@ab reduced BRONJ lesions. (A) Representative images of sockets of rats 4 weeks post‐extraction. The No‐Tx‐ and Gel‐BG‐treated rats had open fistulas, while the Gel‐BG@ab‐treated rats had well‐healed mucosal wounds. No Tx, no treatment. (B) Micro‐CT images showed extraction sockets in the No Tx‐, Gel‐BG‐ and Gel‐BG@ab‐treated rats. (C) The analysis of BV/TV ratio and BMD of the extraction sockets. *n* = 4, *p < 0.05, **p < 0.01, ns indicates no significance. (D) H&E staining of sockets showed necrotic bone and infiltrating immune cells, and the ratio of necrotic bone in the sockets was measured. The Gel‐BG@ab‐treated rats showed less necrotic bone and infiltrating immune cells than that ofthe No Tx‐ and Gel‐ BG‐ treated rats. Scale bars, 200 and 400 μm. (E) Masson's trichrome stained image of sockets showed new bone collagen (blue) increased in the Gel‐BG@ab‐treated rats, and the mature bone tissue or fibrous collagen (red) was much less than that in No Tx‐ and Gel‐BG‐treated rats. Scale bars, 600 μm. **(F)** IHC staining of osterix and OCN in the extraction sockets. The osterix and OCN positive cells in extraction sockets increased in the Gel‐BG@ab‐treated rats. Scale bars, 200 and 400 μm. (G) The ratio of necrotic bone in the extraction sockets was analysed. *n* = 6, **p < 0.01, ns indicates no significance. (H) The percentage of collagen deposition in the extraction sockets was analysed. *n* = 6, **p < 0.01. (I and J) The numbers of osterix^+^ and OCN^+^ osteoblasts were measured in the extraction sockets. *n* = 6, *p < 0.05, **p < 0.01, ns indicates no significance. Data are mean ± SD.

We established a rat model of BRONJ lesions instead of the small tooth extracted socket of the mouse model, and injected the Gel‐BG@ab hydrogel into the bone defects to explore the effect (Figure S4A). The Gel‐BG@ab hydrogel exhibited good soft tissue healing compared with the open fistulas observed in Non Tx‐ and Gel‐BG‐treated rats (Figure 8A and Figure S4B). Some root fragments remained in the extracted tooth sockets, which did not hinder the deposition of newly formed bone [56, 57]. The Gel‐BG@ab hydrogel induced a higher BV/TV ratio and better BMD (Figure 8B,C), fewer dead bone cells and minimal infiltration of inflammatory cells (Figure 8D,G) compared to the No Tx group and Gel‐BG group. Moreover, the Gel‐BG@ab treated rats showed significantly more new blue‐stained bone collagen (Figure 8E,H), and a greater proportion of osterix‐ and OCN‐positive cells (Figure 8F,I,J) than that in the No Tx and Gel‐BG groups. Overall, Gel‐BG@ab promoted osteoblast differentiation and reduced dead bone formation, which was beneficial for alleviating the occurrence and development of BRONJ.

## 4 Discussion

Current management of BRONJ relies mainly on potent antimicrobial therapy and surgical debridement, with limited success [10]. Alterations in immune homeostasis and dysfunction of immune cells are closely associated with BRONJ [58]. γδ T cells, crucial immune surveillance cells, participate in regulating oral mucosal homeostasis [59, 60]. Increased infiltration of γδ T cells in BRONJ lesions has been reported [20]. We found that the release of ZOL from exposed necrotic bone tissues induced the activation and recruitment of γδ T cells. Furthermore, the inhibition of bone formation and bone remodelling is a crucial factor contributing to BRONJ occurrence and development. The inhibition of osteogenic differentiation was alleviated in TCRδ^−/−^ mice, which meant the activated γδ T cells promoted BRONJ by inhibiting bone formation.

To investigate how γδ T cells affected osteoblasts, we screened and verified the significant upregulation of Sema4D in BRONJ lesions. Sema4D is a characteristic signalling protein expressed by T cells [61]. Sema4D decreased significantly with the depletion of γδ T cells, indicating that γδ T cells are an important source of Sema4D in BRONJ lesions. Sema4D was a well‐established factor that inhibited osteoblast differentiation [62]. We observed that sSema4D from γδ T cells could bind to Plexin‐B1/2 on osteoblasts, and the use of anti‐Sema4D antibodies could alleviate the inhibition of osteoblast differentiation.

We screened the downstream pathways of Plexin‐B1/2 through the STRING database and showed the binding form and sites of mTOR and Plexin‐B1/2 by PyMOL. The molecular docking simulation is an important method to find the interaction between two proteins, and a combination of computational validation and experimental validation should be performed after molecular docking. The knockdown of *Plxnb1/2* or the use of the rapamycin (mTOR inhibitor) was performed to observe the effects of ligands on downstream signalling pathways, and the results showed that the inhibition of *Plxnb1/2* decreased the protein levels of p‐mTOR and mTOR. mTOR was a crucial regulatory factor for osteoblast differentiation and bone formation [63]. The use of rapamycin (the inhibitor of mTOR) reversed the inhibition of sSema4D on osteoblast differentiation. In future research, we will explore the interaction mechanism between Plexin‐B1/2 and mTOR through a series of experiments. However, there is a limitation in the experiments; the CO‐IP assays of Plexin‐B1/2 and mTOR were not performed. We will further study in the future.

The Sema4D increased on immune cells after activation, which could be shed from the cell surface by protease [64, 65]. We found that ZOL could enhance the expression of MMPs and increase the cleavage of mSema4D on γδ T cells in vivo and in vitro. MMP3 remained highly expressed in γδ T cells even for a long time of stimulation, and the use of the MMP inhibitors GM6001 and TAPI‐2 inhibited MMP3 activity and alleviated mSema4D shedding. We did not further investigate the mechanism by which ZOL promoted the expression of MMPs in γδ T cells. Whether MMP3 inhibition or knockout in vivo affects the shedding of mSema4D on γδ T cells will be a focus of our future research.

The PPP hydrogel and mesoporous bioactive glass with good biodegradability and biocompatibility are widely used in tissue engineering. Leveraging the bone affinity of bioactive glass (BG) to enhance local retention, combined with the nanocarrier PLGA‐PEG‐PLGA to prolong the half‐life of antibodies and reduce off‐target effects, we developed a Gel‐BG@ab composite hydrogel targeted at Sema4D. This system achieves sustained release and local delivery of anti‐Sema4D antibodies, thereby overcoming the limitations of traditional systemic administration. The released anti‐Sema4D antibodies promoted osteoblast differentiation and reduced the formation of necrotic bone in vivo. Finally, it inhibited the occurrence and progression of BRONJ. Thus, the Gel‐BG@ab composite hydrogel provides new therapeutic approaches for treating BRONJ. This study integrates the functional regulation of γδ T cells with biomaterial engineering, opening up new directions for the precision therapy of BRONJ and providing a theoretical basis and technical references for the immune‐material combination therapy of other bone‐immune related diseases.

## Author Contributions

L.O. and L.S. designed the experiments and supervised the research. S.Q., Z.L., X.T., H.H., Z.Z., R.L., W.Z., Y.Y. and Z.Z. performed the experiments. L.O., S.Q., Z.L., X.T., Z.Z. and W.Z. analysed and interpreted the data. L.O., Y.S., J.H., S.W. and Y.J. wrote or edited the manuscript with input from all coauthors. All authors approved the final version.

## Conflicts of Interest

The authors declare no conflicts of interest.

## Supporting information


**Data S1:** Supporting Information.

## Data Availability

The data that supports the findings of this study are available in the SI of this article.
