# Supplementary material for: Soluble Sema4D From γδ T Cells Exerts Osteoblast Inhibition via Plexin‐B/mTOR Signalling Contributing to Pathogenesis of Bisphosphonate‐Related Osteonecrosis of the Jaws
Source: Cell Prolif. 2025 Sep 4;59(4):e70114. doi: 10.1111/cpr.70114 (PMC13052030; doi:10.1111/cpr.70114)
Supplement: Supplementary file 1 — Data S1: Supporting Information. [file CPR-59-e70114-s001.docx]

**Soluble Sema4D from γδ T cells** **exerts osteoblast inhibition via PlexinB/mTOR signaling contributing to pathogenesis of bisphosphonate-related osteonecrosis of the jaws**

**Supplementary figure legends**

**Supplementary Figure 1 Analysis of γδ T cells in mice.**


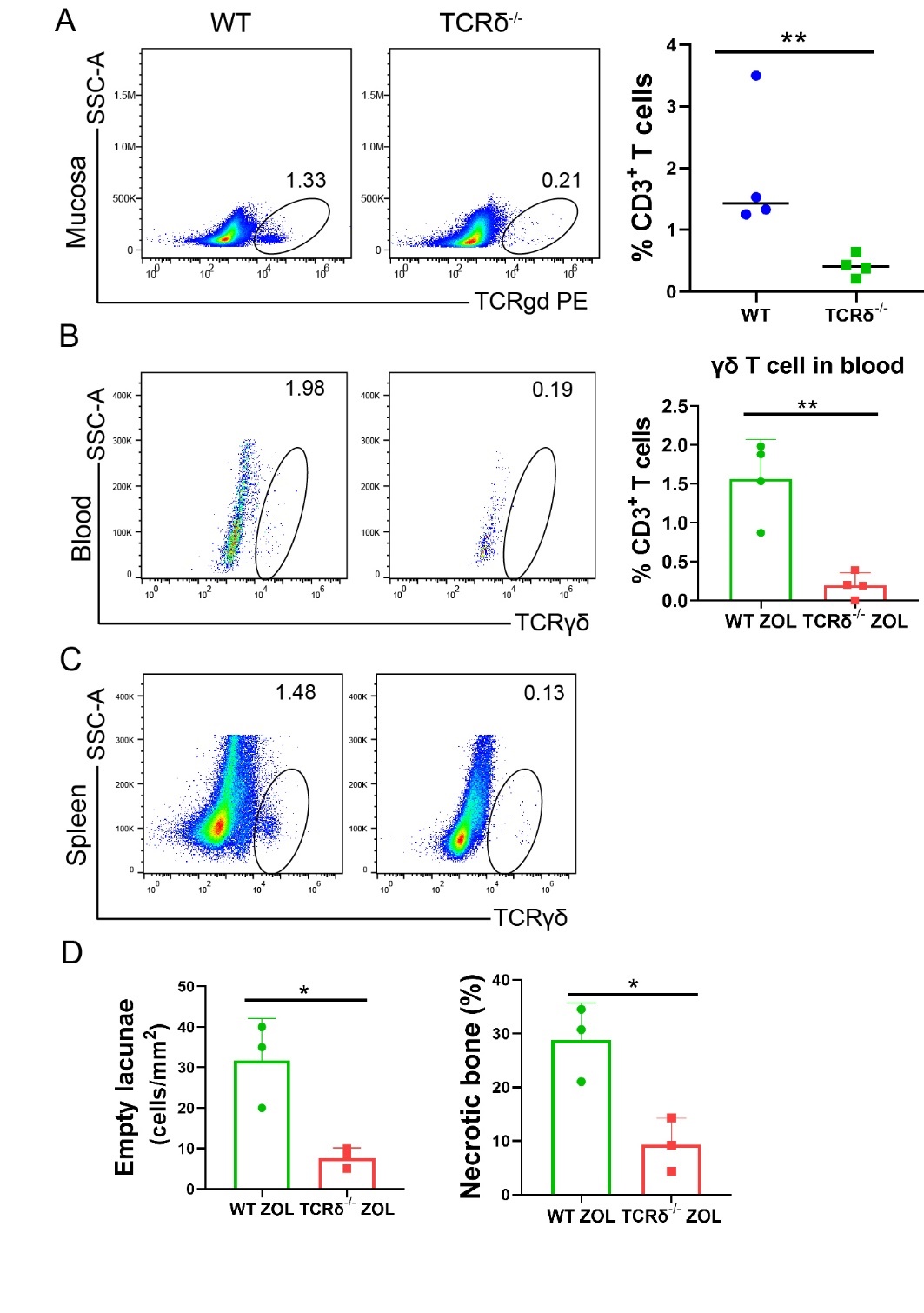


**A**. **B**, **C**. Scatter plots and the percentages of γδ T cells in the mucosa, blood and spleen. n=4, **p < 0.01. **D**. The empty lacunae cell number and the percentage of necrotic bones in extraction sockets were measured. n=3, *p < 0.05. Data are mean ± SD.

**Supplementary Figure 2 Sema4D secreted from activated γδ T cells**


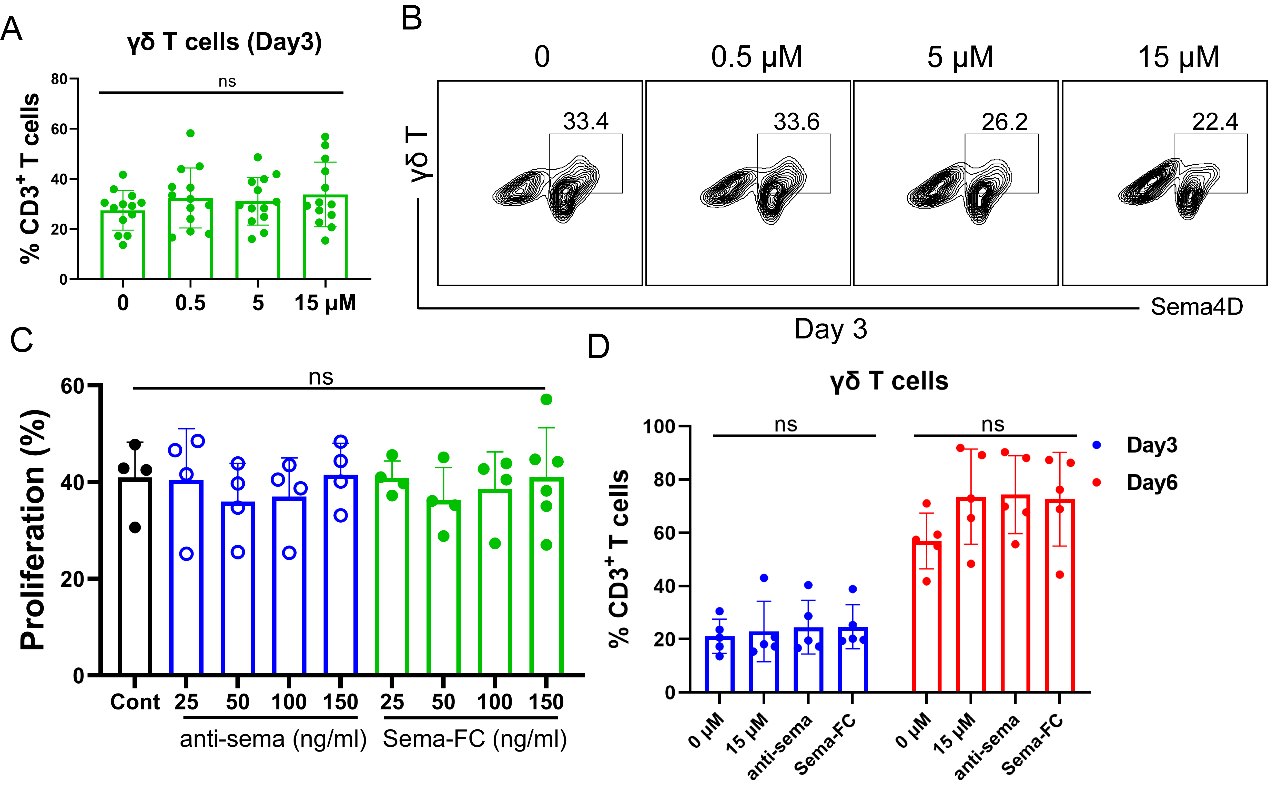


**A**. Purity of γδ T cells incubated with different concentrations of ZOL for 3 days. ns, no significance. **B**. Representative contour plots of Sema4D^+^ γδ T cells incubated with ZOL for 3 days. **C**. Analysis of γδ T cell proliferation after treatment with an anti-Sema4D antibody (αSema4D) and/or Sema4D-Fc stimulation. ns, no significance. **D**. Purity of γδ T cells incubated with an anti-Sema4D antibody (αSema4D) and/or Sema4D-Fc for 3 or 6 days. ns, no significance. Data are mean ± SD.

**Supplementary Figure 3 Sema4D mediated Plexin-B1/2/mTOR signaling**


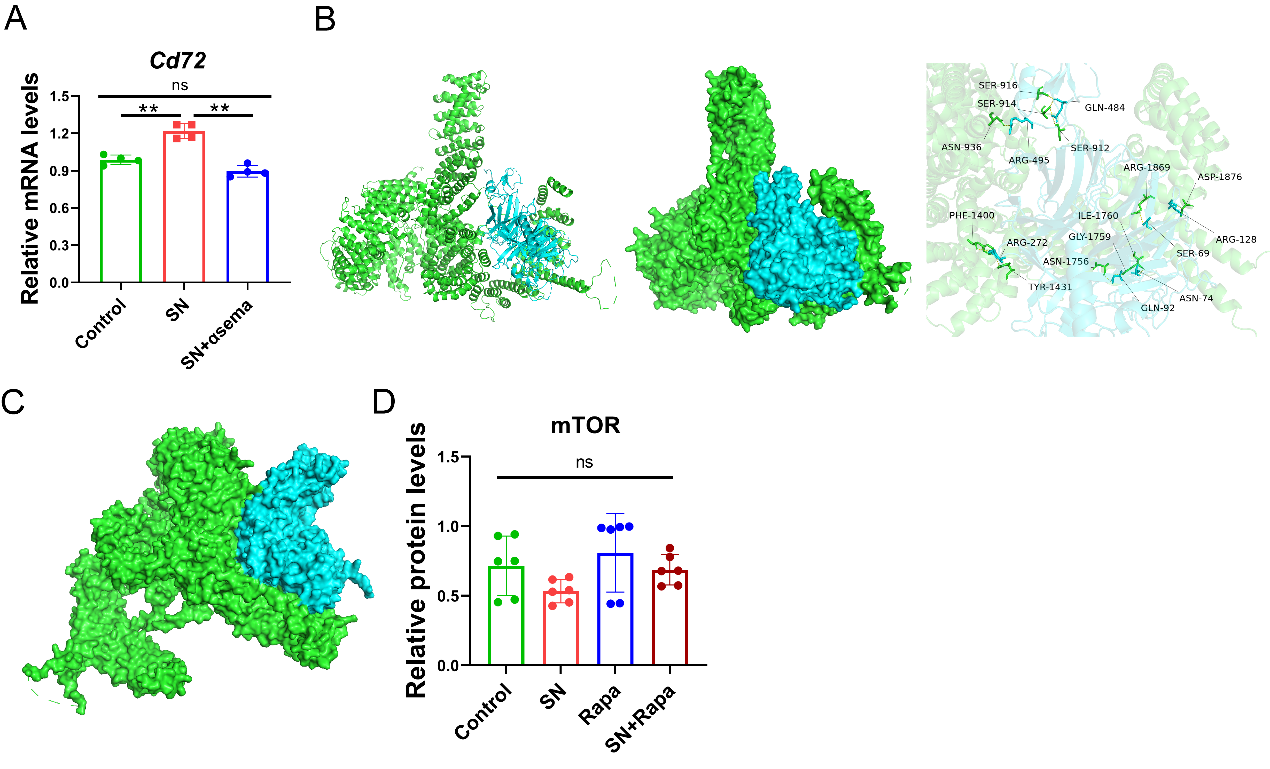


**A**. The mRNA levels of *Cd72* after incubated with the supernatant of γδ T cells and αSema4D. **B**. The binding form and binding sites of mTOR and Plexin B1 were visualized by PyMOL. mTOR protein (Green) and Plexin B1 protein (Blue). **C**. The binding of mTOR to Plexin-B2 was visualized by PyMOL. **D**. Analysis of the protein levels of mTOR after treatment with rapamycin and/or the supernatant. ns, no significance. Data are mean ± SD.

**Supplementary Figure 4 Gel-BG@ab reduced BRONJ lesions**


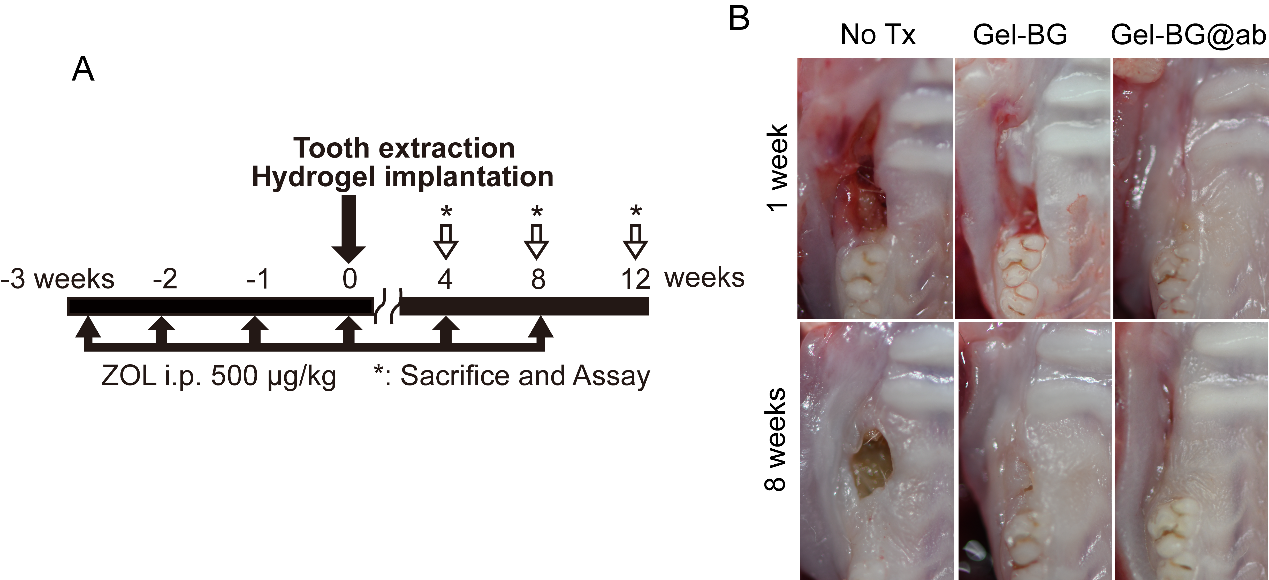


**A**. Schematic illustration of the procedure of Gel-BG@ab treatment. **B**. Representative images of the sockets of mice after tooth extraction for 1 and 8 weeks. The No Tx- and Gel-BG-treated rats had open fistulas, while the Gel-BG@ab treated rats had well-healed mucosal wounds. No Tx, no treatment.

**Supplement table 1 siRNA and RNA primer**

|  | sense | antisense |
| --- | --- | --- |
| siplxnb1-1 | GCUUGCUAAUUACACUCGA(dT)(dT) | UCGAGUGUAAUUAGCAAGC(dT)(dT) |
| siplxnb1-2 | CUACUACUCUUAUGUGGAA(dT)(dT) | UUCCACAUAAGAGUAGUAG(dT)(dT) |
| siplxnb1-3 | GAGCAUCUCUAUGUAGCAA(dT)(dT) | UUGCUACAUAGAGAUGCUC(dT)(dT) |
| siplxnb2-1 | GGCUACCUGUCCACGAAUA(dT)(dT) | UAUUCGUGGACAGGUAGCC(dT)(dT) |
|  |  |  |
| Sema4d | CCTGGTGGTAGTGTTGAGAAC | GCAAGGCCGAGTAGTTAAAGAT |
| Sema3a | CACTGGGATTGCCTGTCTTTT | TGGCACATTGTTCTTTCCGTTT |
| Sema3g | TGCGGCTCTCCTACAGAGAT | GCTTCCCAGAAAGAGGCGAT |
| Sema4a | ACAATGCTACTCACCTCTATGCC | GACCTTGTCTATCAAGATGGGC |
| Sema3e | AGGCTACCGCTGTCACATAAA | GAGCCGTTCTTGATACTCATCC |
| Cd72 | AACGGCAACAATCCAAGAAACC | CCTGACACAATGTCGGCTTTG |
| plxnb1 | CACACATCTACTACACTTGGCAA | CAATCCCGGCTGTCATTCAC |
| plxnb2 | ACCTCCCACCAGTATCCCTTC | GCCTCCCGACACTCGTAGT |
| Alpl | CCAACTCTTTTGTGCCAGAGA | GGCTACATTGGTGTTGAGCTTTT |
| Runx2 | TCCCGTCACCTCCATCCTCTTTC | GAATACGCATCACAACAGCCACAAG |
| Osterix | ACCCCAAGATGTCTATAAGCCC | CGCTCTAGCTCCTGACAGTTG |
| Mmp2 | GGACAAGTGGTCCGCGTAAA | CCGACCGTTGAACAGGAAGG |
| Mmp3 | ACATGGAGACTTTGTCCCTTTTG | TTGGCTGAGTGGTAGAGTCCC |
| Mmp9 | GCAGAGGCATACTTGTACCG | TGATGTTATGATGGTCCCACTTG |
| Mmp11 | GCATTCAGGGGTGATTCAGAC | AGGGCCCCAGAAAGAAATGG |
| Adam17 | GGATCTACAGTCTGCGACACA | TGAAAAGCGTTCGGTACTTGAT |
| Adam10 | ATGGTGTTGCCGACAGTGTTA | GTTTGGCACGCTGGTGTTTTT |
| GAPDH | CCAATGTGTCCGTCGTGGATC | GTTGAAGTCGCAGGAGACAAC |
